# Phytochemical Characterization, Antimicrobial Activity and In Vitro Antiproliferative Potential of *Alchemilla vulgaris* Auct Root Extract against Prostate (PC-3), Breast (MCF-7) and Colorectal Adenocarcinoma (Caco-2) Cancer Cell Lines

**DOI:** 10.3390/plants11162140

**Published:** 2022-08-17

**Authors:** Omer H. M. Ibrahim, Kamal A. M. Abo-Elyousr, Khalid A. Asiry, Nabil A. Alhakamy, Magdi A. A. Mousa

**Affiliations:** 1 Department of Arid Land Agriculture, Faculty of Meteorology, Environment and Arid Land Agriculture, King Abdulaziz University, Jeddah 21589, Saudi Arabia; 2 Department of Pharmaceutics, Faculty of Pharmacy, King Abdulaziz University, Jeddah 21589, Saudi Arabia; 3 Center of Excellence for Drug Research and Pharmaceutical Industries, King Abdulaziz University, Jeddah 21589, Saudi Arabia; 4 Mohamed Saeed Tamer Chair for Pharmaceutical Industries, Faculty of Pharmacy, King Abdulaziz University, Jeddah 21589, Saudi Arabia

**Keywords:** antiproliferative effect, colon cancer, breast cancer, prostate cancer, lady’s mantle

## Abstract

Despite the proven biological activity of the aerial part extract of *Alchemilla vulgaris*, scarce information is available about the activity of the root extract. This encouraged us to initiate the current investigation to study the cytotoxic activity of *A. vulgaris* methanolic root extract against various cancer cell lines in vitro, along with its antimicrobial activity and phytochemical screening. MTT assay was applied to test the cytotoxic effect against the prostate (PC-3), breast (MCF-7) and colorectal adenocarcinoma (Caco-2), together with normal Vero cells. Flow cytometry was employed to assess cell cycle arrest and apoptosis vs. necrosis in PC-3 cells. The expression of apoptosis-related genes (*BAX*, *BCL2* and *P53*) was quantified by qRT-PCR analysis. The obtained results showed strong antiproliferative activity on the three cancer cell lines and the normal Vero cells in a dose-dependent manner. A high selectivity index (SI) was recorded against the three cell lines with PC-3 cells showing the highest SI and the lowest IC_50_. This effect was associated with cell cycle arrest at G1 phase and induction of total apoptosis at 27.18% being mainly early apoptosis. Apoptosis induction was related to the upregulation of the proapoptotic genes *P53* and *BAX* and the downregulation of the antiapoptotic gene *BCL2*. Additionally, the extract demonstrated in vitro antibacterial activity against *Agrobacterium tumefaciens*, *Serratia marcescens* and *Acinetobacter johnsoni*. Additionally, it showed antifungal activity against *Rhizoctonia solani*, *Penicillium italicum* and *Fusarium oxysporium*. Seven phenolic acids and seven flavonoids were detected. The predominant phenolic acids were cinnamic and caffeic acids, while hisperdin and querestin were the principal flavonoids. These findings provide clear evidence about the promising proapoptotic effect of *A. vulgaris* root extract, which contributes to laying the basis for broader and in-depth future investigations.

## 1. Introduction

Phytomedicine research has recently proved a good contribution to the development of multitarget therapy directed principally for the stimulation of defense, protective and repair mechanisms of the body instead of confronting the damaging agents [1,2,3]. Cancer is one of the most common devastating diseases that has a good chance for the application of multitarget therapy using natural plant-based products [4,5]. Lady’s mantle (*Alchemilla vulgaris* Auct. syn., *Alchemilla xanthochlora* Rothm.) is a good example of potential herbal therapeutic agents for many diseases. *A. vulgaris* belongs to the family Rosaceae with a perennial herbaceous growth habit. It is distributed throughout Europe, western Asia and North America [6,7]. *A. vulgaris* is included in the European pharmacopeia and has been reported to contain diverse phytochemical components in aerial and root parts with a variety of biological activities. Phenolic compounds are the predominant compounds identified in previous studies. A limited number of publications is available in previous literature about the phytochemical profile and biological activity of roots of *A. vulgaris*, according to Boroja et al. [3] and Jurić et al. [8]. Their results showed that both extracts of the roots and aerial parts included high content of phenolic compounds especially condensed tannins including ellagic acid and catechin. These components exerted antioxidant, antibacterial, antifungal and anti-inflammatory activities. This supported the idea that *A. vulgaris* is a good source of antioxidant components, as reported by Kovač et al. [6].

The importance of phenolic components is increasingly recognized nowadays through their important role in the oxidation processes. Accordingly, they show several beneficial antimicrobial and anticancer activities [6]. Despite the scarce information available about the anticancer effects of *A. vulgaris*, the previous literature suggested potential anticancer properties revealing promising cytotoxicity against human breast MCF7, ovarian A2780, cervical HeLa and prostate PC-3 cancer cell lines, which was ascribed to its richness in phenolic compounds including catechin, quercetin and its hexoside, luteolin, apigenin, gallic and caffeic acids. The authors ensured that male reproductive cancers, such as prostate cancer, could be targeted by *A. vulgaris* extract, despite the ethnobotanical information about its anticancer potential against female reproductive tissues. Additionally, *A. vulgaris* leaf extract was reported to induce a remarkable reduction in the viability of SH-SY5Y Human neuroblastoma cell line, B16 and B16F10 mouse melanoma cell lines and 4T1 mouse breast cancer followed by loss of dividing potential after 72 h of treatment [9,10]. Nevertheless, more detailed investigations are needed to provide an in-depth explanation of *A. vulgaris* anticancer activity.

This study was, therefore, designed to examine the in vitro antitumor potential of *A. vulgaris* root methanolic extract against PC-3, MCF-7 and Caco-2 cell lines and to provide a deep interpretation of its effects on cell apoptosis, antimicrobial effects and phytochemical profile.

## 2. Materials and Methods

### 2.1. Plant Material and Extract Preparation

Roots of lady’s mantle (*Alchemilla vulgaris* Auct.) were collected during the vegetative growth stage from plants naturally growing in the Wadi Allaqi downstream part of the Egyptian southern desert, Aswan region. The samples were collected in paper bags and directly transported to the Department of Ornamental Plants and Landscape Gardening, Assiut University, Egypt, where they were authenticated by senior staff members of the department. After cleaning with running water followed by distilled water, the roots were cut into small pieces before drying under shade conditions at ~30 °C. After drying, the samples were pulverized and stored in dark containers until used for the preparation of the methanolic extracts. Root powder (100 g) was macerated in MeOH/H_2_O (8:2, *v*/*v*) at a 1: 10 *w*/*v* ratio of sample to solvent and kept on a shaker for three days at room temperature. After filtration, the residues were used to repeat the same process two more times. The combined filtrates from the three times were concentrated under reduced pressure at 50 °C using the rotary evaporator (Hidolph VV2000) to remove methanol. The remaining extract was frozen at −80 °C overnight before subjecting to freeze-drying using Telstar-LyoQuest plus-55 lyophilizer at 1.5 × 10^−4^ mbar for 48 h. The yield of dried extract was estimated (7.15 g) and stored in dark vials at −20 °C. 

### 2.2. HPLC Analysis of A. vulgaris Root Extract

Dry extract (0.1 g) was dissolved in methanol (2 mL) to obtain a sample concentration of 50 mg/mL, which was used for analyzing phenols and flavonoids content. The analysis was performed by HPLC-(Agilent 1100) composed of two LC- pumps pump, a UV/Vis detector and a C18 column (125 mm × 4.60 mm, 5 µm particle size). Chromatograms were obtained and analyzed using the Agilent ChemStation. Phenolic acids were separated by employing a gradient mobile phase of two solvents: Solvent A (Methanol) and Solvent B (Acetic acid in water 1:25), where the detection wavelength was 250 nm. The mobile phase used for the separation of flavonoids was acetonitrile (A) and 0.2% (*v*/*v*) aqueous formic acid (B) with an Isocratic elution (70:30) program. The detection wavelength was set at 360 nm. The gradient program began with 100% B and was held at this concentration for the first 3 min. This was followed by 50% eluent A for the next 5 min, after which the concentration of A was increased to 80% for the next 2 min and then reduced to 50% again for the following 5 min. The detected compounds were matched with standard samples, and their concentrations were calculated from calibration curves to their standard compounds. The final concentrations were expressed as mg/g of dry extract as well as root powder. All standards (chlorogenic acid, catechol, syringenic acid, caffeic acid, ferulic acid, p-coumaric acid, benzoic acid, gallic acid, chlorogenic acid, p-hydroxybenzoic acid, cinnamic acid, salicylic acid, epicatechin, ellagic acid, pyrogallol, protocatechuic acid, tyrosol, 7-OH flavone, naringin, rutin, quercetin, kaempferol, luteolin, hisperdin, catechin) were purchased from Sigma-Aldrich (St. Louis, MO, USA).

### 2.3. Antifungal Activity of A. vulgaris Root Extract

The activity of *A. vulgaris* methanolic root extract was analyzed in vitro against *Rhizoctonia solani*, *Penicillium italicum* and *Fusarium oxysporium*. The PDA (Potato Dextrose Agar) medium was prepared so that it contained various concentrations of *A. vulgaris* extract (from 15.6 to 1000 ppm in two-fold dilutions) in comparison with hymexazol (1000 ppm) as a positive control. After pouring the medium into Petri plates, a 2 mm fungal plug was inoculated in the center of each plate, and then incubated at 28 °C for 10 days. When the fungal growth covered the control (untreated plates), the linear fungal growth was recorded in the control (A) and the treated plates (B).

### 2.4. Antibacterial Activity of A. vulgaris Root Extract

The methanolic extract of *A. vulgaris* roots was tested for its antibacterial activity against *Agrobacterium tumefaciens, Serratia marcescens and Acinetobacter johnsoni* using the agar diffusion test according to the method described by Brulez and Zeller [11]. Briefly, Petri plates filled with NSA (nutrient-sucrose agar) medium were inoculated with different bacteria by spreading the bacterial suspension over the medium surface. After drying, a 9 mm punch was made and filled with the corresponding extract concentration (from 15.6 to 1000 ppm in two-fold dilutions) or amoxicillin (62.5 ppm) as a positive control, and each treatment was replicated four times. The cultures were incubated at 27 °C for two days, after which inhibition zones in mm were recorded for the control (A) and the treatments (B). The growth inhibition percentage was calculated according to Equation (1). The minimum inhibitory concentration (MIC) of the tested extract against the three bacterial strains was determined as the lowest extract concentration that suppresses the bacterial growth after 24 h of incubation:

(1) $$\mathrm{Growth}\;\mathrm{inhibition}=\left(\frac{A-B}{A}\right)\times 100$$

### 2.5. Cytotoxicity Test

#### 2.5.1. Cell Cultures

All cell lines employed in the current study were obtained from the American Type Culture Collection (ATCC, Manassas, VA, USA). Three human cancer cell lines were included, i.e., the prostate (PC-3), breast (MCF-7) and colorectal adenocarcinoma (Caco-2), in addition to normal Vero cells (ATCC CCL-81) isolated from the kidney of *Cercopithecus aethiops*. MCF-7 cells (ATCC HTB-22) were isolated from the mammary gland, breast-derived from pleural effusion metastases, PC-3 cells (ATCC CRL-1435) were isolated from prostate derived from bone metastases and Caco-2 cells (ATCC ATB-37) were isolated from colon tissues. The cells were cultured on RPMI medium 1640 supplemented with fetal bovine serum at 10%, penicillin G at 100 units/mL and streptomycin sulfate at 100 mg/mL. The cultured cells were incubated at 37 °C in a CO_2_ incubator. Cells were harvested by the incorporation with trypsin at 0.25% and EDTA-2Na at 0.025% in PBS (Phosphate-Buffered Saline). 

#### 2.5.2. MTT (3-(4,5-dimethylthiazol-2-yl)-2,5-diphenyltetrazolium bromide) Assay

The MTT assay protocol reported by Bahuguna et al. [12] was applied to test the cytotoxic activity of *A. vulgaris* root extract with slight adaptations. Briefly, cells were harvested from the four investigated cell lines and cultured in the 96-well plates at 100 mL/well (1 × 10^5^ cells/mL) and incubated at 37 °C under CO_2_ conditions. A monolayer of cells was allowed to develop for 24 h before decanting the medium and washing the layer two times with a fresh growth medium. A maintenance medium of RPMI-1640 (Roswell Park Memorial Institute 1640) medium supplemented with 2% fetal bovine serum was used to prepare serial two-fold dilutions (from 31.5 to 1000 ppm) of the extract compared with doxorubicin as a positive control. Any visual signs of cell toxicity were recorded. The plates were incubated for 48 h before conducting the MTT assay. The culture medium was decanted, and 20 mL of MTT solution (5 mg/mL) was added to each well and incubated in the dark for 4 h under the same conditions. The MTT solution was decanted and replaced by DMSO (Dimethyl sulfoxide) to solubilize the formazan crystal (MTT metabolic product) and incubated for 30 min under the same conditions. The optical densities were recorded at 560 nm wavelength for the plated cells using an ELISA reader. The cytotoxicity percentage was estimated from the formula: cytotoxicity percentage = (A_560_ control − A_560_ sample)/A_560_ control × 100. The IC_50_ was calculated for the tested extract against each cell line along with the normal Vero cells, from which the selectivity index (SI) was calculated as SI = IC_50_ calculated for normal cells/IC_50_ calculated for cancer cells. The obtained data were compared with IC_50_ of doxorubicin tested against the same cell lines under the same conditions.

### 2.6. Cell Cycle Arrest Assessment

Since the lowest IC_50_ and the highest SI were recorded for PC-3 cell line, it was employed to examine the cell cycle distribution as per the method previously reported by Alqahtani et al. [13] and Nasr et al. [14]. PC-3 cells were treated with *A. vulgaris* root extract at 88 µg/mL (the IC_50_ recorded against PC-3 cell line) and then incubated for 24 h. The cells were then harvested and washed twice with PBS and fixed with cold 70% ethanol and then stored at 4 °C for 4 h. After that, the fixed cells were rehydrated with PBS and then incubated for 30 min with RNase A at 100 μg/mL, and propidium iodide at 100 μg/mL (ab139418_Propidium Iodide Flow Cytometry Kit/BD, Abcam, Boston, MA, USA). The DNA content was assessed using a flow cytometer (BD FACSCalibur). Propidium iodide fluorescence intensity was collected on FL_2_ of a flow cytometer and 488 nm laser excitation.

### 2.7. Flow Cytometry Assessment of Apoptotic vs. Necrotic Cells

An apoptosis Detection Kit, FITC Annexin V with PI (BioVision, CA, USA) was employed with the manufacturer’s instructions to assess the apoptosis and necrosis in PC-3 cells treated with *A. vulgaris* root extract. PC-3 cells were cultured in 6-well culture plates at 4 × 10^5^ cells/well for 24 h, followed by the treatment with *A. vulgaris* extract at 88 µg/mL and incubated for 24 h. Next, the cells were harvested and washed two times with PBS and resuspended in 100 µL of Annexin V-binding buffer and mixed with 5 μL of each of Annexin V-FITC and propidium iodide dyes. After incubation for 15 min in the dark, apoptotic vs. necrotic cells were assessed using flow cytometry (BD FACSCalibur).

### 2.8. Quantitative Real-Time PCR (qRT-PCR)

The qRT-PCR analysis was conducted for PC-3 cells (4 × 10^5^ cells/mL) in a 6-well plate subjected to the treatment with *A. vulgaris* root extract at 88 µg/mL for 24 h [14]. TRIzol reagent was utilized to extract RNA from both control and treated cells. 1 μg of extracted RNA was converted to cDNA using a BioRad syber green PCR MMX kit. Specific primers of *BAX*, *BCL2* and *P53*, displayed in Table 1, were employed to perform the qRT-PCR using Rotorgene RT- PCR system (Corbett Research, Sydney, Australia). 

### 2.9. Statistical Analysis

All the experiments were carried out in three independent trials and data were presented as mean ± standard deviation. Statistix software (ver. 8.1, Analytical Software, Tallahassee, FL, USA) was used to perform the statistical analysis. One-way ANOVA was applied with LSD to compare the group means. 

## 3. Results

### 3.1. Chemical Composition of A. vulgaris Root Extract

**Figure 1:**
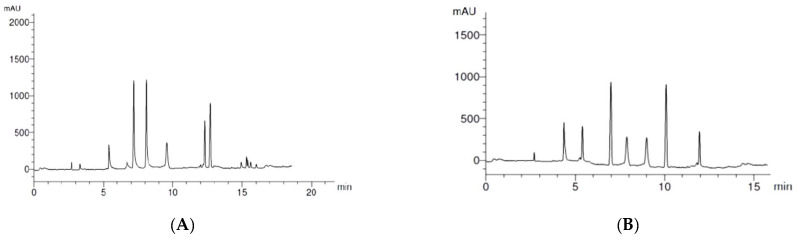
HPLC chromatograms of *Alchemilla vulgaris* root extract: (**A**) phenol components; (**B**) flavonoid components.

Quantitative analysis of phenolic and flavonoid components of *A. vulgaris* methanolic root extract was conducted using HPLC. A total of seven phenolic acids were identified, as shown in Table 2 and Figure 1A, with a total concentration of 93.9 μg/g root powder. The predominant phenolic acid was cinnamic and caffeic acids (25.8 and 24.7 μg/g root powder, respectively). The other detected acids were in the following descending order: salicylic (13.7 μg/g), ellagic (12.1 μg/mg), syringenic (7.6 μg/g), gallic (6.8 μg/g) and protocatchuic (3.2 μg/g) acids, in addition to three more unknown compounds

A total of seven flavonoids were detected with a total concentration of 70.2 μg/g root powder. Both hisperdin and querestin recorded the highest concentrations (16.5 and 14.7 μg/g, respectively), as shown in Table 3 and Figure 1B. These were followed in descending order by Catechin (11.0 μg/g), Naringin (9.1 μg/g), Rutin (7.4 μg/g), Kampferol (6.0 μg/g) and Luteolin (5.5 μg/g). 

### 3.2. Antimicrobial Activity of A. vulgaris Root Extract

Antibacterial activity of *A. vulgaris* root extract showed significant growth inhibition of the three tested bacteria including the human pathogenic Gram-positive bacterium *Acinetobacter johnsonii*, the bioagent Gram-positive bacterium *Serratia marcescens* and the plant pathogenic Gram-negative bacterium (Table 4). The lower three concentrations (15.6, 31.3 and 62.5 µg/mL) showed no growth inhibition for any of the three bacteria. The inhibitory effect of *A. vulgaris* root extract started at 125 µg/mL (MIC) for the three bacterial strains, achieving a 7.67, 8.00 and 8.00 mm inhibition zone, corresponding to 27.78, 3.33 and 33.33% inhibition percentages for *Serratia marcescens*, *Acinetobacter johnsonii* and *Agrobacterium tumefaciens*, respectively. Higher concentrations exhibited a dose-dependent increase in the inhibition zone and growth inhibition percentage. Growth of *Acinetobacter johnsonii* showed the strongest inhibition percentage, whereas both *Serratia marcescens* and *Agrobacterium tumefaciens* responded similarly. Meanwhile, *Serratia marcescens* was the most affected by the antibiotic positive control (Amoxicillin), then *Serratia marcescens*, followed by *Acinetobacter johnsonii* and then *Agrobacterium tumefaciens*. 

*A. vulgaris* root extract at all the tested concentrations exhibited dose-dependent suppression of the growth of the employed fungal strains: *Rhizoctonia solani, Penicillium italicum* and *Fusarium oxysporium* (Table 5). This indicates that the MIC for the three fungal species is higher than 1000 µg/mL. The strongest inhibition effect of the extract was noticed against *Fusarium oxysporium*, recording 45.56% growth inhibition at 1000 µg/mL extract compared with 81.85% inhibition in response to the hymexazol positive control treatment. 

### 3.3. Antiproliferative Activity of A. vulgaris Root Extract

**Figure 2:**
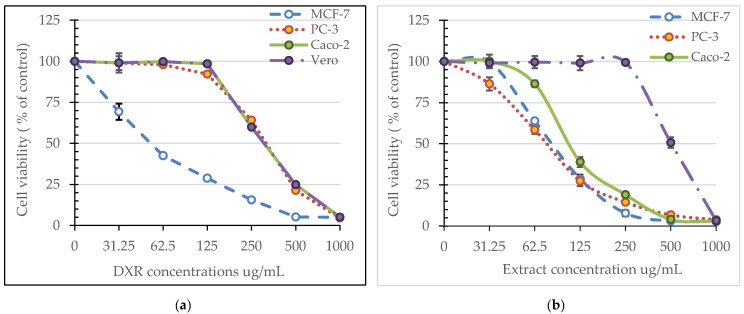
MTT assay results on viability/cytotoxicity of MCF-7, PC-3, Caco-2 and Vero cells: (**a**) effect of *A. vulgaris* root extract; (**b**) effect of doxorubicin. Values are represented as the mean (*n* = 3) ± SD.

**Figure 3:**
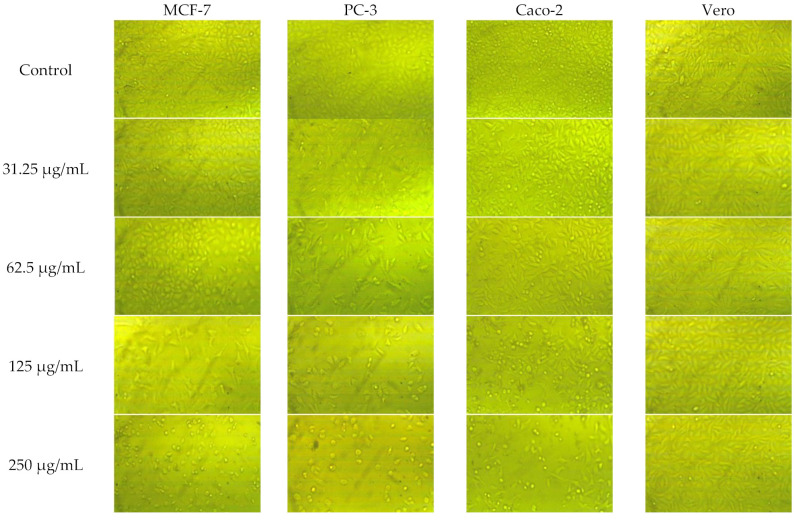

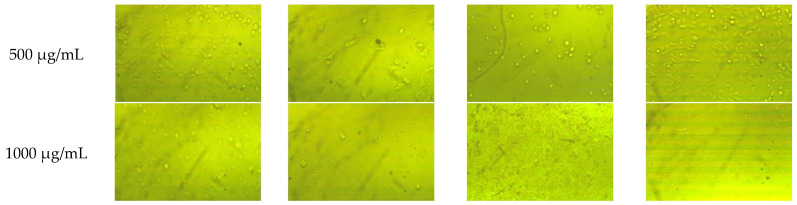
Morphology of MCF-7, PC-3, Caco-2 and Vero cells after 48 h from the treatment with different concentrations of *A. vulgaris* root extract. Pictures were taken using a Reichert-Jung inverted biological microscope, series 1820 Biostar with a 10× objective coupled with MEM1300 microscope USB digital camera.

The methanolic extract of *A. vulgaris* roots showed a cytotoxic effect on the prostate (PC-3), breast (MCF-7) and colorectal adenocarcinoma (Caco-2), together with normal Vero cells in a dose-dependent manner (Figure 2). Morphological characteristics of the treated and untreated cells are clear in the photos presented in Figure 3. Noticeably high selectivity indices (SI) were recorded especially in the case of PC-3 and Caco-2 cell lines (6.69 and 5.36) reaching ≈6 folds of these recorded by doxorubicin (1.03 and 1, respectively) as shown in Table 6. The lowest IC_50_ and the highest SI were recorded for PC-3 cell line, and thus, it was employed in further studies on cell cycle arrest, proapoptosis and the expression of the apoptosis-related genes.

### 3.4. Effect of A. vulgaris Root Extract on Cell Cycle Arrest in PC-3 Cells

**Figure 4:**
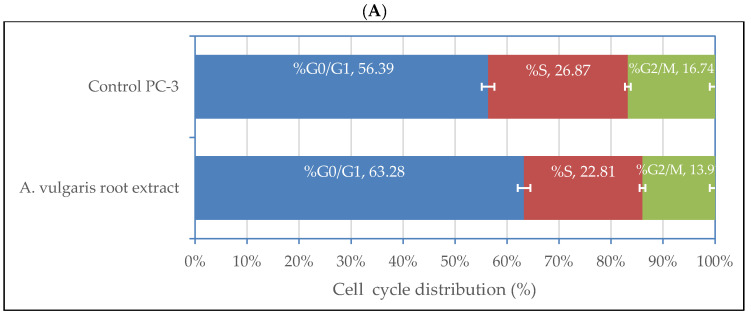

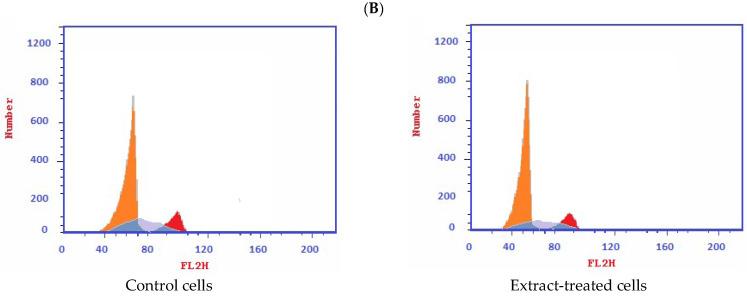
Cell cycle distribution of PC-3 cells treated with *A. vulgaris* root extract at 88.0 µg/mL: (**A**) quantitative cell cycle distribution percentage illustrated by the bar graph with cell growth arrest@ G1; (**B**) flow cytometry histogram showing DNA content of extract-treated cells. The data presented are the means (*n* = 3) ± SD indicated by the horizontal bars.

Cell cycle arrest was studied in PC-3 cells exposed to *A. vulgaris* root extract at 88 µg/mL and the data are illustrated in Figure 4. The extract treatment caused cell cycle arrest at G1 phase. Assessment of cell cycle distribution and DNA content of the cells revealed higher G0/G1 in treated PC-3 cells (63.28%) compared with the control (56.39%). The proportion of other cell phases showed fewer percentages than that in the control cells.

### 3.5. Effect of A. vulgaris Root Extract on Apoptosis and Necrosis of Cells

**Figure 5:**
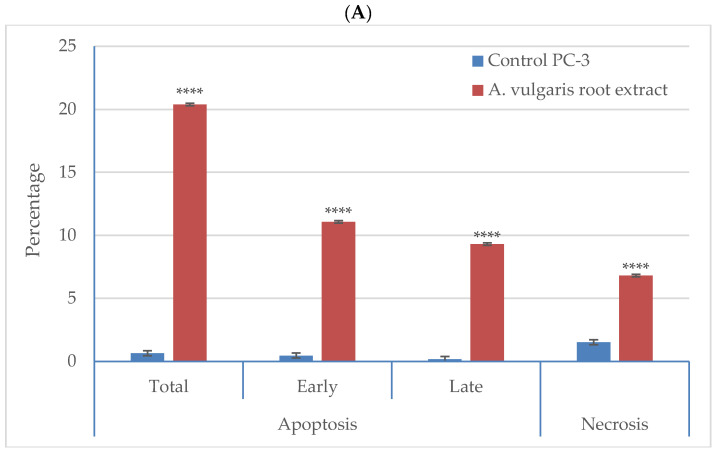

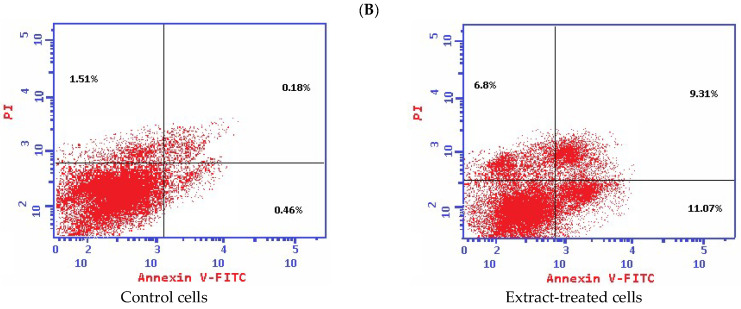
Apoptotic effect of *A. vulgaris* root extract at 88.0 µg/mL on PC-3 cells: (**A**) percentages of the total, early and late apoptotic and necrotic cells in extract-treated PC-3 cells compared with the control cells; (**B**) flow cytometry dot plots of extract-treated cells showing necrotic cells (upper left quadrant), late apoptotic cells (upper right quadrant), viable cells (lower left quadrant) and early apoptotic cells (lower right quadrant). Significance differences between treated and control cells were determined using unpaired *t*-test, **** *p* < 0.0001. The data presented are the means (*n* = 3) ± SD indicated by the vertical bars.

Exposing PC-3 cells to *A. vulgaris* root extract at 88 µg/mL induced considerably higher apoptosis than the control cells (Figure 5). The total apoptosis in treated cells reached 20.38%, including 11.07% early apoptosis and 9.13% late apoptosis. These values correspond to about 31 times the recorded apoptosis percentage in the control cells (0.64%). On the other hand, treated PC-3 cells recorded only 6.8% of necrotic cells compared with 1.51% necrosis in the control cells.

### 3.6. Effect of A. vulgaris Root Extract on the Expression of Apoptosis-Related Genes in PC-3 Cells

**Figure 6:**
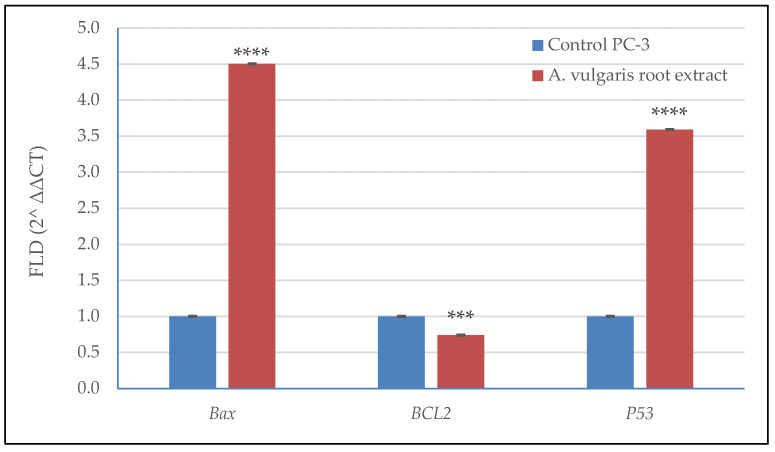
Expression of *BAX*, *BCL2* and *P53* genes in PC-3 cells treated with *A. vulgaris* root extract at 88.0 µg/mL using qRT-PCR analysis. Significance differences between treated and control cells were determined using unpaired *t*-test, *** *p* < 0.001, **** *p* < 0.0001. The data presented are the means (*n* = 3) ± SD indicated by the vertical bars.

qRT-PCR analysis was employed to quantify the expression of three apoptosis-related genes (*BAX*, *BCL2* and *P53*) in PC-3 cells exposed to treatment with *A. vulgaris* root extract at 88 µg/mL (Figure 6). A considerable decrease was recorded in the antiapoptotic *BCL2* gene (0.74% of the control), while the expression of the proapoptotic genes *P53* and *BAX* showed a considerable increase recording 450% and 360% of that in the control. Upregulation of the proapoptotic genes *P53* and *BAX* helps explain the mechanism of apoptosis induction in response to *A. vulgaris* root extract treatment. 

## 4. Discussion

The methanolic extract of *A. vulgaris* roots investigated in the current study exhibited strong antiproliferative activity on the prostate (PC-3), breast (MCF-7) and colorectal adenocarcinoma (Caco-2), together with normal Vero cells in a dose-dependent manner. A high selectivity index was recorded for the tested extract against the three cell lines with PC-3 cells showing the highest SI and the lowest IC_50_. This effect was associated with cell cycle arrest at the G1 phase and induction of apoptosis at 20.38%, being mainly early apoptosis. Apoptosis induction was connected to the upregulation of the proapoptotic genes *P53* and *BAX* and the downregulation of the antiapoptotic gene *BCL2*. Induction of proapoptotic by *A. vulgaris* root extract suggests the need for the inclusion of other cancer cell lines in further broader and in-depth studies since apoptosis is considered a potential pathway for the development of new drugs [15,16]. Unlike aerial parts extract, the root extract of *A. vulgaris* has been investigated in a limited number of previous studies, including Boroja et al. [3] and Jurić et al. [8]. However, the cytotoxicity previously reported for the extract of the aerial parts supports the promising activities revealed by root extract in our study. *A. vulgaris* leaf extract was reported to induce a remarkable reduction in viability of SH-SY5Y human neuroblastoma cell line, B16 and B16F10 mouse melanoma cell lines and 4T1 mouse breast cancer followed by loss of dividing potential after 72 h of treatment [9,10]. Similar results were obtained by Vlaisavljević et al. [17] against human breast MCF7, ovarian A2780, cervical HeLa and prostate cancer PC-3 cell lines. These studies provided supportive findings to those exerted by ours regarding the strong antiproliferative effect of *A. vulgaris* root extract against the prostate cancer cell line (PC-3). The authors [17] ensured that male reproductive cancers such as prostate cancer could be targeted by *A. vulgaris* extract despite the ethnobotanical information about its anticancer potential against female reproductive tissues. Jurić et al. [8] stated that methanol extracts of aerial parts and roots of *A. vulgaris* prevented cisplatin-induced hepatorenal and testicular toxicity in rats through mitigation of the level of several parameters related to the cisplatin-induced oxidative stress and the serum of liver, kidney and testicle injury and tissue morphology. According to Jelača et al. [9,10], the mechanism of cell death induced by *A. vulgaris* is through the occurrence of autophagy after the treatment, as indicated by the presence of autophagosomes in melanoma B16 cells by flow cytometry, whereas a certain percentage of melanoma B16F10 cells was subjected to programmed cell death in a caspase-independent manner. Additionally, Moqidem [18] suggested that cell death induced by *A. vulgaris* on SH-SY5Y human neuroblastoma cell line was through apoptosis induction accompanied by significant downregulation of the cell cycle genes (CDK4, CDK6 and E2F3), and upregulation of the tumor suppressor gene PTEN.

*A. vulgaris* root extract demonstrated in vitro antibacterial activity against the human pathogenic Gram-positive bacterium *A. johnsonii*, the bioagent Gram-positive bacterium *S. marcescens,* and the plant pathogenic Gram-negative bacterium *A. tumefaciens*. Additionally, it showed antifungal activity against *R. solani, P. italicum* and *F. oxysporium.* The strongest inhibition effect of the extract was induced against *A. johnsonii* bacterial strain and *F. oxysporium* fungal strain. The calculated MIC for the three bacterial strains was 125 µg/mL, while it was higher than 1000 µg/mL for the fungal ones. Accordingly, *A. vulgaris* root extract is considered a potent antibacterial agent based on the classification suggested by Kuete [19] considering substances as potent, moderate or weak antimicrobial agents if they induced antibacterial activity with MICs below 0.1 mg/mL, between 0.1 and 0.625 or above 0.625 mg/mL, respectively. High antibacterial activity was also reported by Boroja et al. [3] against *Enterococcus faecalis*, *Salmonella typhimurium*, *Micrococcuslysodeikticus* and *Bacilus mycoides* as the most sensitive examined bacterial species to the aerial parts and root extracts of *A. vulgaris*. They also recorded antifungal activity for the roots extract against *Phialophora fastigiate, Penicillium canescens*, *Trichoderma viride*, *T. longibrachiatum*, *Aspergillus glaucus* and *Fusarium oxysporum*. According to Boroja et al. [3], their study was the first to deal with the antimicrobial activity of *Alchemilla* roots. Their results revealed similar antimicrobial activity and anti-inflammatory effects of aerial and root extracts with a higher antioxidant activity of root extract. Our findings, together with those of the published studies, provide evidence with respect to the biological activity of *A. vulgaris* root extract. 

The promising antiproliferative activity, apoptosis induction and antimicrobial activity induced by *A. vulgaris* root extract could be discussed in the context of its content of phenolics and flavonoids analyzed by HPLC. Seven phenolic acids and seven flavonoids were detected. The predominant phenolic acids were cinnamic and caffeic acids, while hisperdin and querestin were the principal flavonoids. The other phenolic acids identified included salicylic, ellagic, syringenic, gallic and protocatchuic acids. Flavonoids, as well, comprised catechin, naringin, rutin, kampferol and luteolin. The strong biological activities of phenolic compounds were extensively reported by numerous previous authors, such as Vlaisavljević et al. [17], who ascribed the promising cytotoxicity of *A. vulgaris* extract against human breast MCF7, ovarian A2780, cervical HeLa and prostate cancer PC-3 cell lines to its richness in phenolic compounds including catechin, quercetin and its hexoside, luteolin, apigenin, gallic and caffeic acids. Phenolics are among the important active secondary metabolites with reported biological activities on human physiological functions, which led to recognition as dietary supplements and remedies in complementary medicine [8]. Their role is ascribed mainly to their antioxidant effects and thus chemoprevention and chemotherapeutic effects. Moqidem [18] signified the role of phenolic compounds detected abundantly in *A. vulgaris* extract in the reduction of SH-SY5Y cells viability. Antioxidant activity of flavonoid and phenolic components detected in *A. vulgaris* extract also has a significant contribution to its biological, including cytotoxic, activity. Jain et al. [20] recorded high flavonoid and phenolic content of methanolic and ethyl acetate extract of *A. vulgaris*, which led to further potent radical scavenging results with methanolic extract exerting higher antioxidant activity. Quercetin is one of the flavonoids detected in *A. vulgaris*, which has been reported to mitigate cisplatin-induced nephrotoxicity and hepatotoxicity [21,22]. Jurić et al. [8] reported the presence of more than 20 different phenolic compounds in aerial and root extracts of *A. vulgaris* analyzed by UHPLC/DAD, where ellagic acid, catechin and catechin gallate were dominant components in both extracts. Despite the higher number of phenolic compounds compared to our findings, their results indicated that kaempferol and quercetin were under detection limit in the methanolic root extract, while they were found in the extract of the aerial parts. These results contradict another published study by Vlaisavljević et al. [17], who reported only 11 phenolic compounds in aerial parts extract of *A. vulgaris* analyzed by the LC-MS/MS technique, with the absence of both kaempferol and quercetin. Variation in the phytochemical profile between the aerial and root parts of *A. vulgaris* and between plant material collected from different sources is expected and sensible based on the known effects of environmental factors on plant growth and induction of secondary metabolites. Additionally, there is a clear effect for different techniques of chromatographic analysis employed in the published literature.

## 5. Conclusions

In the context of the scarce information available about the detailed biological activity of *A. vulgaris* roots extract, the obtained findings from the current investigation are of special importance. The methanolic extract of *A. vulgaris* roots exhibited strong antiproliferative activity on PC-3, MCF-7 and Caco-2 in addition to Vero cells in a dose-dependent manner, with PC-3 cells showing the highest selectivity index and the lowest IC_50_. This effect was associated with cell cycle arrest at the G1 phase and induction of apoptosis through the upregulation of the proapoptotic genes *P53* and *BAX* and the downregulation of the antiapoptotic gene *BCL2*. Additionally, it showed potent antibacterial and antifungal activity in vitro. These effects were connected to the phytochemical constituents of the studied extract, especially phenolic acids and flavonoids.

## Figures and Tables

**Table 1:** Specific primers of *BAX*, *BCL2* and *P53* used to perform qRT-PCR analysis for PC-3 cells in response to *A. vulgaris* root extract treatment.

Gene	Primer
*BAX*	F: 5’-ATGGACGGGTCCGGGGAG-3’
	R: 5’-ATCCAGCCCAACAGCCGC-3’
*BCL2*	F: 5’-AAG CCG GCG ACGACT TCT-3’
	R: 5’-GGT GCC GGT TCA GGTACT CA-3’
*P53*	F: 5’-ATGTTTTGCCAACTGGCCAAG -3’
	R: 5’-TGAGCAGCGCTCATGGTG-3’

**Table 2:** Concentrations of phenol components of *Alchemilla vulgaris* root extract screened by HPLC.

No	Compound	RT * min	Concentration μg/mL	µg/g Dry Extract	µg/g Root Powder
1	Unknown	2.8	NA **	NA	NA
2	Unknown	3.4	NA	NA	NA
3	Syringenic acid	5.1	5.33	106.6	7.6
4	Cinnamic acid	7.0	18.05	361	25.8
5	Caffeic acid	8.0	17.26	345.2	24.7
6	Gallic acid	9.7	4.79	95.8	6.8
7	Salicylic acid	12.3	9.56	191.2	13.7
8	Ellagic acid	12.9	8.49	169.8	12.1
8	Unknown	15.0	NA	NA	NA
10	Protocatchuic acid	15.5	2.21	44.2	3.2
11	Unknown	15.7	NA	NA	NA
12	Unknown	16.0	NA	NA	NA

* RT = retention time, ** NA = not applicable.

**Table 3:** Concentrations of flavonoid components of *Alchemilla vulgaris* root extract by HPLC.

No	Compound	RT * min	Concentration μg/mL	µg/g Dry Extract	µg/g Root Powder
1	Unknown	2.8	NA **	NA	NA
2	Naringin	4.4	6.33	126.6	9.1
3	Rutin	5.2	5.14	102.8	7.4
4	Querestin	7.0	10.28	205.6	14.7
5	Kampferol	7.9	4.23	84.6	6.0
6	Luteolin	9.1	3.88	77.6	5.5
7	Hisperdin	10.0	11.56	231.2	16.5
8	Catechin	12.0	7.69	153.8	11.0

* RT = retention time, ** NA = not applicable.

**Table 4:** Antibacterial activities of *A. vulgaris* root extract against *Serratia marcescens, Acinetobacter johnsonii* and *Agrobacterium tumefaciens*. Data are shown as mean ± SD, *n* = 3.

Extract Concentrations (µg/mL)	Inhibition Zone (mm)			Growth Inhibition (%)		
	*Seratia*	*Acinetobacter*	*Agrobacterium*	*Seratia*	*Acinetobacter*	*Agrobacterium*
15.6	6.00 ± 0.00	6.00 ± 0.00	6.00 ± 0.00	0.00	0.00	0.00
31.3	6.00 ± 0.00	6.00 ± 0.00	6.00 ± 0.00	0.00	0.00	0.00
62.5	6.00 ± 0.00	6.00 ± 0.00	6.00 ± 0.00	0.00	0.00	0.00
125	7.67 ± 0.47	8.00 ± 0.00	8.00 ± 0.34	27.78	33.33	33.33
250	8.00 ± 0.82	8.33 ± 0.94	7.67 ± 0.46	33.33	38.89	27.78
500	10.33 ± 0.46	11.33 ± 0.47	9.33 ± 0.44	72.22	88.89	55.56
1000	11.00 ± 0.04	12.33 ± 0.40	11.00 ± 0.82	83.33	105.56	83.33
Negative control	6.0 ± 0.00	6.00 ± 0.00	6.0 ± 0.00	0.00	0.00	0.00
Amoxicillin (62.5 ppm)	41.3 0.40	38.33 ±1.25	37.0 ± 84	588.89	538.89	516.67
LSD (0.05)	0.81	1.19	0.93	-	-	-

(-) = not performed

**Table 5:** Antifungal activities of *A. vulgaris* root extract against *Rhizoctonia solani*, *Penicillium italicum* and *Fusarium oxysporium*. Data are shown as mean ± SD, *n* = 3.

Extract Concentrations (µg/mL)	Mycelial Growth (mm)			Growth Inhibition (%)		
	*Rhizoctonia*	*Penicillium*	*Fusarium*	*Rhizoctonia*	*Penicillium*	*Fusarium*
15.6	8.23 ± 0.05	8.30 ± 0.08	6.17 ± 0.12	8.52	7.78	31.48
31.3	8.23 ± 0.05	8.10 ± 0.08	5.70 ± 0.08	8.52	10.00	36.67
62.5	7.87 ± 0.09	7.83 ± 0.24	5.63 ± 0.09	12.59	12.96	37.41
125	7.87 ± 0.05	7.60 ±0.28	5.50 ± 0.14	12.59	15.56	38.89
250	7.37 ± 0.07	7.30 ±0.18	5.17 ± 0.12	18.15	18.89	42.59
500	7.37 ± 0.12	7.30 ±0.16	5.23 ± 0.09	18.15	18.89	41.85
1000	7.00 ± 0.22	6.97 ± 0.05	4.90 ± 0.08	22.22	22.59	45.56
Negative control	9.0 ± 0.00	9.0 ± 0.00	9.0 ± 0.00	0.00	0.00	0.00
Hymexazol (1000 ppm)	1.8 ± 0.24	2.9 ± 0.69	1.6 ± 0.12	79.63	68.15	81.85
LSD (0.05)	0.26	0.58	0.22	-	-	-

(-) = not performed

**Table 6:** IC_50_ (µg/mL) and selectivity index of *A. vulgaris* root extract against different cell lines.

	IC_50_ (µg/mL)				Selectivity Index		
	Vero	MCF-7	PC-3	Caco-2	MCF-7	PC-3	Caco-2
*A. vulgaris* root extract	592.47	92.25	88.60	110.51	6.42	6.69	5.36
Doxorubicin	35.09	5.40	34.11	35.09	6.50	1.03	1.00

## References

[1] Tadić V.M., Krgović N., Ana Ž. (2020). Lady’s mantle (*Alchemilla vulgaris* L., Rosaceae): A review of traditional uses, phytochemical profile, and biological properties. Nat. Med. Mater..

[2] Castro-Puyana M., Pérez-Sánchez A., Valdés A., Ibrahim O.H.M., Suarez-Álvarez S., Ferragut J.A., Micol V., Cifuentes A., Ibáñez E., García-Cañas V. (2017). Pressurized liquid extraction of *Neochloris oleoabundans* for the recovery of bioactive carotenoids with anti-proliferative activity against human colon cancer cells. Food Res. Int..

[3] Boroja T., Mihailović V., Katanić J., Pan S.P., Nikles S., Imbimbo P., Monti D.M., Stanković N., Stanković M.S., Bauer R. (2018). The biological activities of roots and aerial parts of *Alchemilla vulgaris* L.. South Afr. J. Bot..

[4] Bhattacharya B., Akram M., Balasubramanian I., Tam K.K.Y., Koh K.X., Yee M.Q., Soong R. (2012). Pharmacologic synergy between dual phosphoinositide-3-kinase and mammalian target of rapamycin inhibition and 5-fluorouracil in PIK3CA mutant gastric cancer cells. Cancer Biol. Ther..

[5] Abdul-Hafeez E.Y., Orabi M.A.A., Ibrahim O.H.M., Ilinskaya O., Karamova N.S. (2020). In vitro cytotoxic activity of certain succulent plants against human colon, breast and liver cancer cell lines. South Afr. J. Bot..

[6] Kovač M.J., Jokić S., Jerković I., Molnar M. (2022). Optimization of deep eutectic solvent extraction of phenolic acids and tannins from *Alchemilla vulgaris* L.. Plants.

[7] Duckstein S.M., Lotter E.M., Meyer U., Lindequist U., Stintzing F.C. (2012). Phenolic constituents from *Alchemilla vulgaris* L. and *Alchemilla mollis* (Buser) Rothm. at different dates of harvest. Z. für Nat. C.

[8] Jurić T., Katanić Stanković J.S., Rosić G., Selaković D., Joksimović J., Mišić D., Stanković V., Mihailović V. (2020). Protective effects of *Alchemilla vulgaris* L. extracts against cisplatin-induced toxicological alterations in rats. South Afr. J. Bot..

[9] Jelača S., Drača D., Dajić Stevanović Z., Mijatović S., Jovanović I., Jovanović M., Jurišević M., Arsenijević N., Maksimović-Ivanić D. Antitumor potential of *Alchemilla vulgaris* L. in ortotopic mouse breast cancer model. Proceedings of the 6th European Congress of Immunology.

[10] Jelača S., Drača D., Dajić Stevanović Z., Jovanović I., Pavlović S., Gajović N., Mijatović S., Arsenijević N., Maksimović-Ivanić D. Multiple effects of *Alchemilla vulgaris* L. extract on melanoma cells and tumor microenvironment. Proceedings of the 6th European Congress of Immunology.

[11] Brulez W., Zeller W. (1981). Seasonal changes of epiphytic *Erwinia amylovora* on ornamentals in relation to weather conditions and course of infections. Acta Hortic..

[12] Bahuguna A., Khan I., Bajpai V.K., Kang S.C. (2017). MTT assay to evaluate the cytotoxic potential of a drug. Bangladesh J. Pharmacol..

[13] Alqahtani S.A., Nasr F.A., Noman O.M., Farooq M., Alhawassi T., Qamar W., El-Gamal A. (2020). Cytotoxic Evaluation and Anti-Angiogenic Effects of Two Furano-Sesquiterpenoids from *Commiphora myrrh* Resin. Molecules.

[14] Nasr F.A., Noman O.M., Alqahtani A.S., Qamar W., Ahamad S.R., Al-Mishari A.A., Alyhya N., Farooq M. (2020). Phytochemical constituents and anticancer activities of *Tarchonanthus camphoratus* essential oils grown in Saudi Arabia. Saudi Pharm. J..

[15] Bremer E., Van Dam G., Kroesen B.J., de Leij L., Helfrich W. (2006). Targeted induction of apoptosis for cancer therapy: Current progress and prospects. Trends Mol. Med..

[16] Pfeffer C.M., Singh A.T.K. (2018). Apoptosis: A target for anticancer therapy. Int. J. Mol. Sci..

[17] Vlaisavljević S., Jelača S., Zengin G., Mimica-Dukić N., Berežni S., Miljić M., Stevanović Z.D. (2019). *Alchemilla vulgaris* agg.(Lady’s mantle) from central Balkan: Antioxidant, anticancer and enzyme inhibition properties. RSC Adv..

[18] Moqidem Y. (2021). Evaluation of the Anticancer Potential of Alchemilla vulgaris Extract Against Human Neuroblastoma Cells.

[19] Kuete V. (2010). Potential of Cameroonian plants and derived products against microbial infections: A review. Planta Med..

[20] Jain S., Yadav A.S., Gothalwal R. (2021). Assessment of total phenolic, flavonoid content and in vitro antioxidant properties of *Alchemillia vulgaris* (lady’s mantle). J. Adv. Sci. Res..

[21] Sanchez-Gonzalez P.D., Lopez-Hernandez F.J., Perez-Barriocanal F., Morales A.I., Lopez-Novoa J.M. (2011). Quercetin reduces cisplatin nephrotoxicity in rats without compromising its anti-tumour activity. Nephrol. Dial. Transplant..

[22] Verma P.K., Raina R., Prawez S., Sultana M., Singh M., Kumar P. (2018). Protective mechanisms of quercetin on cisplatin induced oxidative damage in hepatic tissue of wistar rats. Proc. Natl. Acad. Sci. India Sect. B Biol. Sci..

